# Mapping Thrombosis Serum Markers by ^1^H-NMR Allied with Machine Learning Tools

**DOI:** 10.3390/molecules29245895

**Published:** 2024-12-13

**Authors:** Lucas G. Martins, Bruna M. Manzini, Silmara Montalvão, Millene A. Honorato, Marina P. Colella, Gabriela G. Y. Hayakawa, Erich V. de Paula, Fernanda A. Orsi, Erik S. Braga, Nataša Avramović, Folurunsho Bright Omage, Ljubica Tasic, Joyce M. Annichino-Bizzacchi

**Affiliations:** 1 Laboratory of Biological Chemistry, Department of Organic Chemistry, Institute of Chemistry, Universidade Estadual de Campinas, Campinas 13083-862, SP, Brazil; lgmartins1984@gmail.com (L.G.M.); e264601@dac.unicamp.br (E.S.B.); bright@unicamp.br (F.B.O.); 2 Laboratory of Hemostasis, Hemocentro-Unicamp, Universidade Estadual de Campinas, Campinas 13083-878, SP, Brazil; brunamanzini@gmail.com (B.M.M.); silmara@unicamp.br (S.M.); millene.ealhonorato@gmail.com (M.A.H.); marinasp@unicamp.br (M.P.C.); hayakawa@unicamp.br (G.G.Y.H.); erich@unicamp.br (E.V.d.P.); ferorsi@unicamp.br (F.A.O.); 3 University of Belgrade, Faculty of Medicine, Institute of Medical Chemistry, 11000 Belgrade, Serbia; natasa.avramovic@med.bg.ac.rs

**Keywords:** thrombosis, metabonomics, antiphospholipid syndrome (APS), antiphospholipid antibodies (aPLs), venous thromboembolism (VTE), machine learning

## Abstract

Machine learning and artificial intelligence tools were used to investigate the discriminatory potential of blood serum metabolites for thromboembolism and antiphospholipid syndrome (APS). ^1^H-NMR-based metabonomics data of the serum samples of patients with arterial or venous thromboembolism (VTE) without APS (n = 32), thrombotic primary APS patients (APS, n = 32), and healthy controls (HCs) (n = 32) were investigated. Unique metabolic profiles between VTE and HCs, APS and HCs, and between VTE and triple-positive APS groups were indicative of the significant alterations in the metabolic pathways of glycolysis, the TCA cycle, lipid metabolism, and branched-chain amino acid (BCAA) metabolism, and pointed to the complex pathogenesis mechanisms of APS and VTE. Histidine, 3-hydroxybutyrate, and threonine were shown to be the top three metabolites with the most substantial impact on model predictions, suggesting that these metabolites play a pivotal role in distinguishing among APS, VTE, and HCs. These metabolites might be potential biomarkers to differentiate APS and VTE patients.

## 1. Introduction

Antiphospholipid syndrome (APS) is an acquired immune-mediated thrombophilia with clinical presentations that include venous, arterial, or microvascular thrombosis and pregnancy morbidities [1,2,3]. Thrombosis recurrence is frequently observed in APS [2]. The diagnosis of APS includes one clinical criterion and the presence of antiphospholipid antibodies (aPL), such as lupus anticoagulant (LAC), anticardiolipin, and/or anti-β2 glycoprotein I [3]. aPL directed to phospholipid-binding proteins on endothelial cells, monocytes, and platelets’ surfaces activates a procoagulant and inflammatory state leading to pro-inflammatory and thrombotic phenotypes [4,5,6,7,8]. The identification of APS patients with a higher risk of the first or a recurrence of a thrombotic episode is a challenge in many clinical situations. The indication of continuous anticoagulation in patients with APS and unprovoked arterial thrombosis or VTE is well-defined, but long-term thromboprophylaxis is controversial for APS patients with provoked VTE or without a previous thrombotic episode [4]. High-risk conditions for thrombotic recurrence include positive LAC, triple positivity for aPL, or a history of arterial and venous thrombosis [5]. In this sense, research efforts are currently seeking alternative blood biomarkers that are capable of improving patient diagnosis, treatment, and stratification, identifying those patients that may benefit from different treatment schedules, particularly in thrombosis, and extended anticoagulation [6].

Metabonomics using NMR can be applied to qualitatively and quantitatively characterize biological samples. NMR metabonomics shows some important advantages such as an almost direct analysis of complex liquid samples, which favors its performance in samples derived from whole blood, such as serum and plasma [7,8]. Thus, metabonomics, in an untargeted, unbiased analysis, allows us to understand the molecular underpinnings of the disease [8], aiming to characterize and quantify all the small molecules with molecular masses lower than 1.5 kDa that are measurable in serum samples of patients [9]. Studies using metabolomics strategies [9,10,11] pointed to changes in some important metabolic pathways, including carbohydrate, lipid, and amino acid metabolism immediately after thrombosis. Studies using mass spectrometry reported decreased acylcarnitines in the blood samples of patients with DVT and in those with a high risk of PE [10,11]. Differences between metabolomic plasma profiles associated with the long-chain acylcarnitine family were described when 40 unprovoked VTE patients were compared with age-matched controls [11]. Previously, we compared the metabolomic profiles of 40 VTE patients and 40 healthy individuals using NMR and observed differences, especially in the spectral regions that correspond to glucose, lipids, unsaturated lipids, and glycoprotein A. Comparative analysis pointed to altered ratios of glucose/lactate and branched-chain amino acids (BCAAs) to alanine, which might be associated with the fingerprints of thrombosis. These alterations are detectable even months after the acute episode [12]. All these studies provided evidence that metabolomics might be important for revealing biomarkers in VTE and stratifying the risks of patients with this condition [12].

Therefore, applying NMR-based metabonomics, we investigated the serum metabolic profiles of the thrombotic patient groups with and without APS compared with HCs. Our goal was to identify serum metabolites that might distinguish the groups, helping us to understand the biology involved in the disease.

## 2. Results

The groups were nominated as thrombotic APS, VTE (thromboembolic disease without APS), and HCs (healthy controls), and all clinical details are presented in Tables S1 and S2 (Supplementary Information). In the VTE and HC groups, most of the participants were women. In the APS group, the majority were men. The median age of the APS participants was the highest compared with the other groups (Table S1). Four ethnic groups were identified in the study: Caucasians, Afro-descendants, Indigenous, and Asians. Moreover, the presence of comorbidities and clinical conditions was observed. Around 53.3% of participants in the HC group were overweight, and 46.9% were obese in the VTE group. The use of medications was observed, and antihypertensive drugs were the most commonly reported. Patients with arterial events were included: four (12.5%) in the APS group and one (3.1%) in the VTE group. According to the risk factors for VTE, they were considered as provoked in 7 (25%) in the APS group and 20 (64.5%) in the VTE patient group. Mild transitory risk factors were observed in 5/7 (71.4%) and 13/20 (65%) of the APS and VTE groups, respectively. Major transitory risk factors were observed in 1/7 (14.3%) and 5/20 (25%) of the APS and VTE groups, respectively. Persistent risk factors were observed in 1/7 (14.3%) and 2/20 (10%) of the APS and VTE groups, respectively. The proximal lower limbs were the site most reported, with 14 (43.8%) and 19 (59.4%) in the APS and VTE groups, respectively. PE was observed in 7 (21.9%) APS patients and in 14 (43.8%) VTE patients. Warfarin was the main therapy chosen for both the APS and VTE groups and was maintained in 28 (87.5%) and 2 (6.3%) of them, respectively. Twenty-nine APS patients (87.5%) and two VTE patients (6.3%) were on lifetime anticoagulation with warfarin, and those with previous arterial thrombosis were also treated with aspirin. D-dimer was analyzed in patients on and not on anticoagulation therapy. VTE participants under anticoagulation treatment (n = 16, 50.0%) had median D-dimer values of 291.0 mg/dL (IQR = 170.0–606.8), and in patients without anticoagulation therapy (n = 15, 46.9%), median D-dimer values of 333.0 mg/dL (IQR = 202.5–467.3) were recorded. The APS patients on anticoagulation therapy (n = 30, 93.8%) showed median values of 239.8 mg/dL (IQR = 151.0–332.4). Only one APS patient was not on anticoagulation therapy (3.1%) and presented a D-dimer value of 312.00 mg/dL. There was no significant difference between the groups on anticoagulation therapy in the VTE and APS patient groups (*p* = 0.4155). The presence of aPL was investigated among all participants. As expected, the VTE and HC groups did not present any antibodies. The aPL profile in the APS group showed that 28 (87.5%) presented with lupus anticoagulant (LAC), 12 (37.5%) with LAC plus anti-beta 2 glycoprotein I, 7 (21.9%) with anticardiolipin (aCL), and 8 (25.0%) were triple positive (Table S2). Natural anticoagulants like protein C, protein S, and antithrombin were evaluated in samples from all patients. The results showed that all values were within the normal range. Factor V Leiden was present in heterozygosity in one (3.1%) VTE patient and two APS patients (6.3%). The mutation G20210A in the prothrombin gene was heterozygous in one (3.1%) VTE patient and two (6.3%) APS patients.

**Figure 1:**
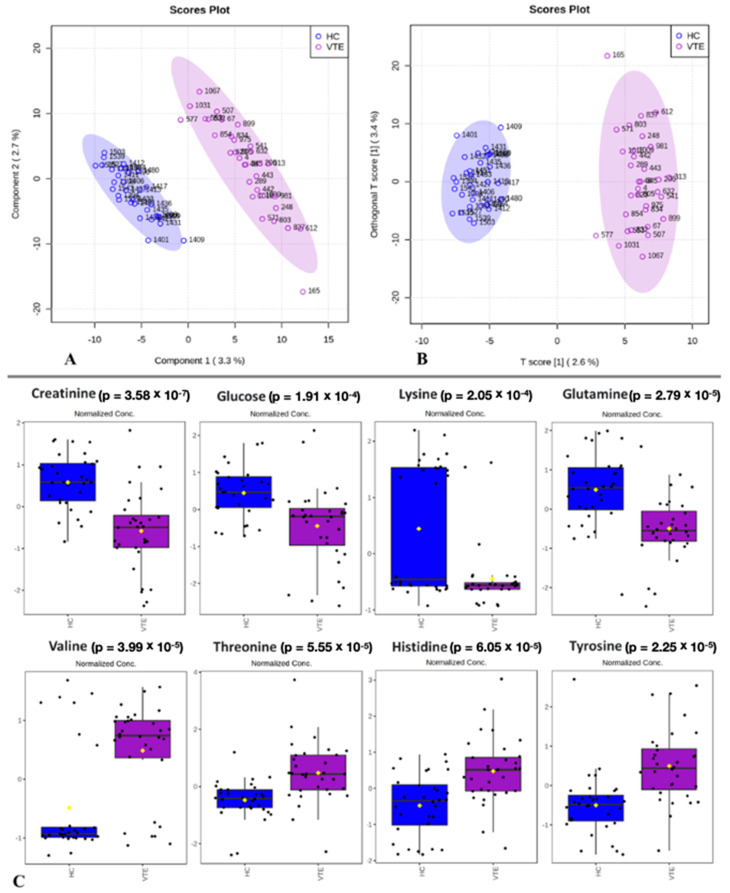
Metabolomics using NMR data for VTE (purple) and HC (blue) serum samples. (**A**) PLS-DA scores in PC 1 vs. PC 2 for CPMG data (accuracy: 0.80, R^2^: 0.97, Q^2^: 0.44). (**B**) oPLS-DA scores (R^2^Y: 0.97 and Q^2^: 0.41). (**C**) Boxplots of the main serum metabolites that were found to be different between the investigated groups, VTE (purple) and HCs (blue). Each box contains the metabolite variation according to a univariate *t*-test analysis. Box plots show groups’ means (yellow diamonds) and medians (horizontal black lines) within a box of 50% of the data.

**Figure 2:**
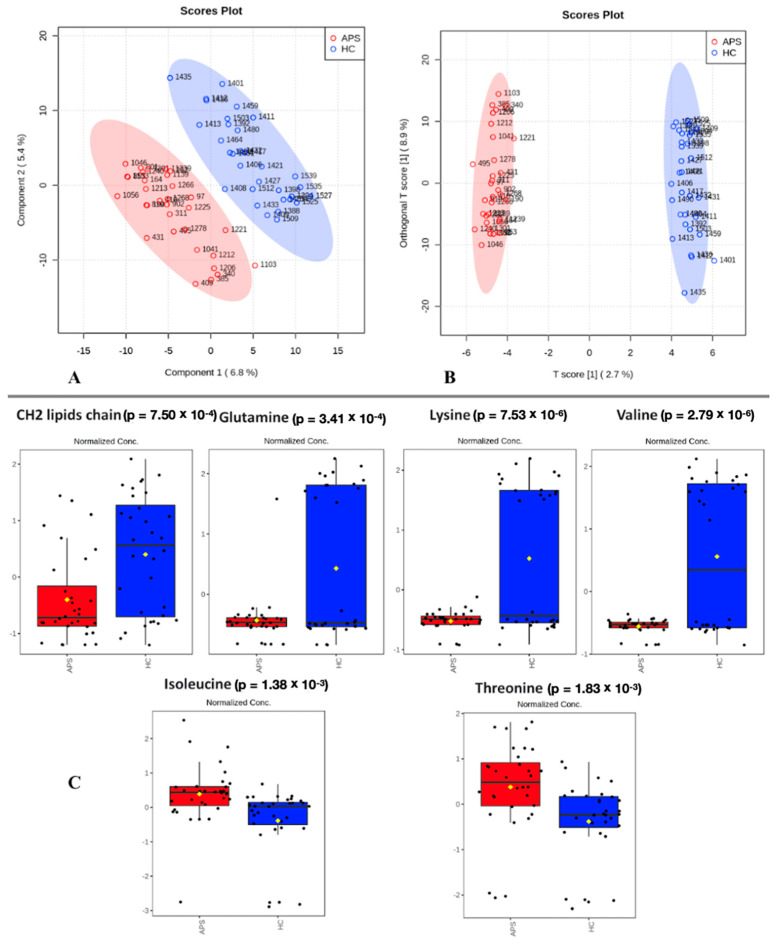
Metabolomics using NMR for APS patients (red) and HCs (blue) according to the ^1^H-NMR of sera. (**A**) PLS-DA scores in PC 1 vs. PC 2 on CPMG data (accuracy: 0.87, R^2^: 0.99, Q^2^: 0.42). (**B**) oPLS-DA scores (R^2^Y: 0.99 and Q^2^: 0.45). (**C**) Boxplots of the main serum metabolites that were found to be different between the investigated groups, APS patients (red) and HCs (blue). Each box contains the metabolite variation according to a univariate *t*-test analysis. Box plots show groups’ means (yellow diamonds) and medians (horizontal black lines) within a box of 50% of the data.

**Figure 3:**
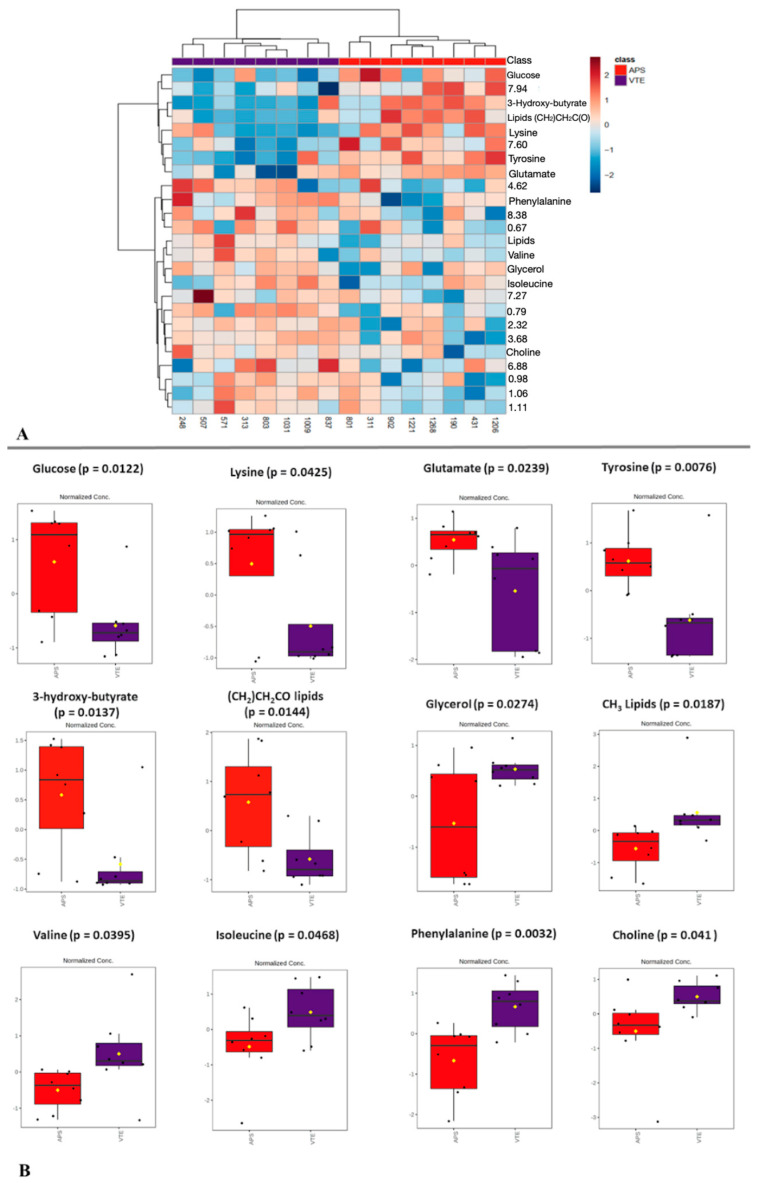
Metabolomics using NMR in VTE (purple) and APS (triple positive, in red) serum samples. (**A**) Clustering results are shown as a heatmap (distance measure using Euclidean distance and a clustering algorithm using Ward’s method) for the APS (red) and VTE (purple) serum samples. (**B**) Variation in the identified serum metabolites that distinguished between APS (triple positive) and VTE-matched individuals. Box plots show groups’ means (yellow diamonds) and medians (horizontal black lines) within a box of 50% of the data.

The metabolomics data are shown in Figure 1, Figure 2 and Figure 3, and serum fingerprints (1D and 2D NMR spectral data, Figure S1) confirmed the significant spectral differences in the VTE and APS groups compared with HCs (Figure S1). VTE and APS samples showed differences in 16 metabolites compared with HCs (Table S3).

The PLS-DA and oPLS-DA results for VTE and HCs showed the inherent grouping within samples, and determining variables that distinguish these groups were identified (Figure 1). The variable importance in projection (VIP) scores in the univariate *t*-test analysis (Table S4) identified different concentrations of eight metabolites in the VTE and HC groups (Figure 1C) with reduced concentrations of creatinine, glucose, lysine, and glutamine and increased concentrations of valine, threonine, histidine, and tyrosine in VTE.

Likewise, the APS group was distinguished from the HC group, as presented in Figure 2. The VIP scores combined with the univariate *t*-test analysis (Table S5) determined six important metabolites that differed in concentration among the analyzed groups, with decreased concentrations of -CH_2_- from lipids, valine, lysine, and glutamine and increased concentrations of isoleucine and threonine with, *p*-values < 0.002 in APS (Figure 2C).

Comparing the VTE and APS groups (n = 32 for each group) using analog chemometrics failed to identify a statistically significant difference between the groups; hence, machine learning methods were implemented. However, there was a subgroup (n = 8) named the triple-positive APS group due to the presence of the three antiphospholipid antibodies, who was matched by gender and age with the other eight patients from the VTE group [13]. Clustering and *t*-test analyses were performed for these groups to evaluate the cluster formations (Table S6). Heatmap variables were identified (*p* < 0.05), with metabolites indicated on the right (Figure 3A), and the metabolites’ boxplots from the *t*-test are shown in Figure 3B.

The investigated APS patient group showed higher amounts of glucose, lysine, glutamate, tyrosine, 3-hydroxy-butyrate, and methylene from lipids, while the VTE patient group showed higher amounts of glycerol, lipids, valine, isoleucine, phenylalanine, and choline. To ensure that no false positive results were reported, ROC (receiver operating characteristic curve) analysis was performed for each metabolite indicated in Figure 3B. The ROC curves are presented in Figure S2, from which it is possible to see the selectivity and specificity of each metabolite. The curves presented an excellent AUC (area under the curve, AUC > 0.8) for most metabolites and outstanding AUCs (AUC > 0.9) for phenylalanine and -CH_3_ from lipids. Just the lysine ROC curve presented an AUC of <0.7.

In a comparison of the three groups, the HC (healthy controls), VTE (venous thromboembolism), and APS (antiphospholipid syndrome) groups, classifications were achieved through a univariate analysis of metabolite levels, with significant differences identified using the Kruskal–Wallis test followed by the Benjamini–Hochberg false discovery rate (BH-FDR) correction for multiple comparisons. Additionally, ROC AUC values were computed to evaluate the diagnostic potential of each metabolite by comparing the HC group with the VTE group, the HC group with the APS group, and the VTE group with the APS group. Consequently, Table 1 lists the metabolites with a q-value ≤ 0.05, indicating statistical significance after BH-FDR correction. The table provides details on the direction of the change (increase or decrease), raw *p*-values, q-values, and AUC ROC values for the comparisons: HCs vs. VTE, HCs vs. APS, and VTE vs. APS.

Notably, glucose and isoleucine showed a decrease in concentrations (*p* < 0.05), correlating with the progression of the disease states, both displaying average AUC values above 0.6, indicating their potential diagnostic relevance (Table 1). Other metabolites, including tyrosine, histidine, glutamate, threonine, and the ketone body 3-hydroxybutyrate, were observed to increase in concentration with disease progression, suggesting altered metabolic states associated with these conditions (Table 1).

To find the most appropriate machine learning (ML) method, we first performed supervised multiclass classification on the three classes (VTE, APS, and HCs) using four common multiclass classifiers: random forest, MLP, XGBoost, and the support vector machine. According to the assessment conducted, the random forest classifier showed the highest classification power, with accuracy and F1-score of 62% (Figure 2). The clinical relevance of the ML classification is presented using additional metrics that may better indicate the efficacy of the model (Table 2).

The random forest and XGBoost classifiers demonstrated equivalent performance across accuracy, F1-score, and recall, all achieving a score of 0.62, and a precision of 0.63 and 0.61, respectively, indicating a balanced classification capability across the three classes. However, the random forest classifier outperformed XGBoost in terms of the ROC AUC, with a score of 0.82 compared with 0.76, respectively, suggesting better overall model discrimination. The MLP and support vector classifiers showed lower performance across all metrics, with MLP achieving an accuracy of 0.45 and SVM being even lower at 0.41, indicating challenges in capturing the complex patterns within the dataset for these models (Table 3).

The random forest classifier showed high precision (0.89) and recall (0.80) for the VTE class, leading to a strong F1-score of 0.84, indicative of reliable performance in identifying VTE cases. For the HC class, the classifier achieved balanced precision and recall (both 0.56), resulting in a moderate F1-score. The APS class had the lowest scores, with a precision of 0.45 and a recall of 0.50, yielding an F1-score of 0.48, reflecting challenges in accurately classifying APS cases, as previously shown in the chemometric analysis.

**Figure 4:**
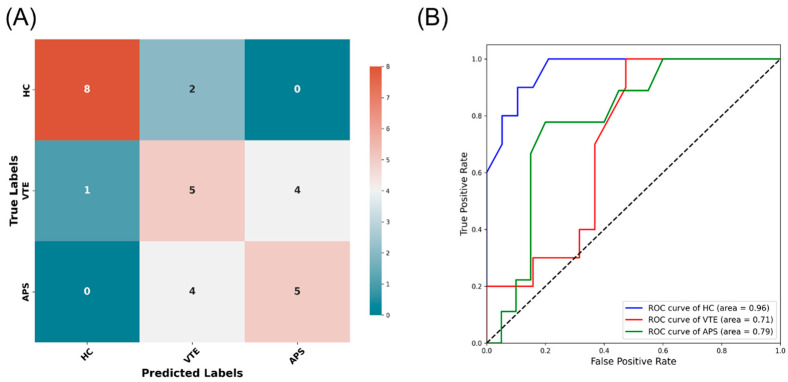
(**A**) Confusion matrix of random forest’s multiclass classification performance. The number of true labels (actual classes) is displayed along the vertical axis, while the predicted labels (predicted classes) are displayed along the horizontal axis. Each cell in the matrix represents the count of samples from the expected class (row) predicted to be in a certain class (column), with the diagonal cells indicating correct predictions. (**B**) Receiver operating characteristic (ROC) curves for multiclass classification. The ROC curves depict the diagnostic ability of the multiclass classification model for each class: healthy controls (HCs), venous thromboembolism (VTE), and antiphospholipid syndrome (APS).

The model exhibited excellent discriminative performance for the HC class (AUC = 0.96) as shown in Figure 4, indicating a high true positive rate and a low false positive rate. The ROC curve for VTE showed an AUC of 0.71. For APS, the AUC was 0.79. The classifier demonstrated substantial discriminatory ability between the HC and VTE classes, with 8 out of 10 HC cases correctly identified and 5 out of 9 VTE cases accurately classified. However, discernment between VTE and APS proved more challenging, with misclassification of four VTE cases as APS. Likewise, the model struggled to distinguish APS cases accurately, with an equal split of correct classifications and misclassifications (five each), indicating potential overlaps in the feature space or insufficient model complexity to capture the nuances between these two classes, as shown in the chemometric analysis.

**Figure 5:**
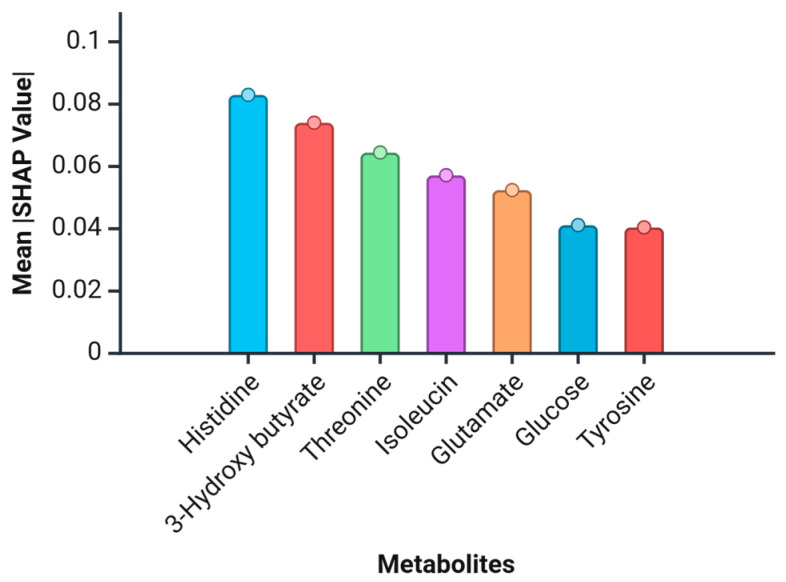
Mean SHAP values for the metabolites. This bar chart displays the mean SHAP values for the seven metabolites contributing to the multiclass classification model’s decisions. SHAP (SHapley Additive exPlanations) values quantify the impact of each feature on the model’s prediction, with higher values indicating a greater influence. Metabolites are ordered by their mean SHAP value, highlighting the most significant contributors to the model’s output.

Histidine, 3-hydroxybutyrate, and threonine are the top three metabolites with the most substantial impact on model predictions (Figure 5), suggesting that these metabolites play a pivotal role in distinguishing among APS, VTE, and HCs. Histidine’s prominent position indicates a potential biomarker of interest, possibly due to its role in protein structure and metabolism. Isoleucine, glutamate, glucose, and tyrosine also show significant SHAP values (Figure 5), underscoring their importance in the classification process to a lesser extent than the top three.

## 3. Discussion

Multivariate statistical analysis results clearly showed the separation of VTE and HCs, APS and HCs, and the triple-positive APS and matched-VTE groups, respectively. The changes in the metabolites indicated important alterations in the metabolic pathways of glycolysis, the TCA cycle, lipid metabolism, and branched-chain amino acid (BCAA) metabolism. Significant alterations in the metabolomic profiles pointed to the complex pathogenesis mechanisms of APS and VTE.

Creatinine, glucose, lysine, glutamine, valine, threonine, histidine, and tyrosine were the most affected metabolites distinguishing between the VTE and control groups. Decreased concentrations of creatine, glucose, glutamine, and lysine in the VTE group indicated altered glycolysis and glutaminolysis compared with HCs. Maekawa et al. showed that increased lactate in the jugular model of venous thrombosis pointed to active glycolysis of the main thrombus of the erythrocytes [14]. Our group recently studied patients with deep venous thrombosis (DVT) compared with the controls, confirming the change in glucose/lactate and branched amino acids (BCAAs) concerning alterations in the glycolysis and BCAA metabolic pathways [12]. On the other hand, Obi et al. provided NMR-based metabolomic data in old mice with induced thrombus compared with young control mice showing increased concentrations of glutamine, phenylalanine, and proline [8]. Namely, increased concentrations of these three metabolites were linked to vein wall weight and vein wall P-selectin levels based on the fact that the aging activity of enzymes that catalyzed their conversion decreased higher oxidative stress [15]. On the other hand, increased concentrations of valine, threonine, histidine, and tyrosine in the VTE group compared with the HC group are correlated to alterations of energy or amino acid metabolism [16]. Amino acids are involved in energy production and protein synthesis [17]. Especially, increased concentrations of BCAAs are crucial regulators of platelet activation and are a risk factor for arterial thromboembolism [17]. To the best of our knowledge, up to now, BCAA dysfunction is correlated only with arterial risk, but not with VTE risk [9,17]. In our recent study of the metabolomic profiles of the DVT group compared with controls, we determined changes in leucine, valine, and alanine, as in these present results [10], providing the correlation of BCAA with venous thrombosis for the first time. Sung et al. found reduced concentrations of TCA metabolites and increased concentrations of carnitine, sphingomyelins, phosphatidylcholine, and triglycerides in DVT mice [6], pointing to a disturbed TCA cycle explained by the reduced availability of acetyl-CoA, which is correlated with disturbed fatty acid metabolism, including carnitine metabolites. Regarding increased levels of ceramide, Sung et al. explained these under the assumption that they are responsible for the simulation of endothelial cell activation and triggering thrombus formation [6].

A comparison of the NMR-based metabolomic profiles of the APS and HC groups indicated six significantly different concentrations of metabolites. Increased concentrations of isoleucine and threonine in the APS group compared with the controls confirmed the importance of amino acid metabolism, which was altered like in the VTE group (Figure 1). On the other hand, decreased concentrations of -CH2- from lipids, valine, lysine, and glutamine pointed to altered lipid metabolism, amino acid metabolism, and glutaminolysis (Figure 2). Zhang et al. [18] explored LC-MS-based metabolomic profile changes between women with APS who had experienced recurrent abortion and controls, showing five potential biomarkers (uric acid, creatine, creatinine, arginine, and thyroid hormones) that are closely linked to APS. Recently, we reported some metabolomic alterations in APS patients with deep venous thrombosis (DVT) compared with healthy individuals, highlighting enhanced glycolysis with glucose consumption and lactate production, as well as alterations in some aromatic acids [19]. These metabolite changes also pointed to alterations in energy metabolism and aromatic acid metabolism.

The increased concentrations of glucose, lysine, glutamate, tyrosine, 3-hydroxy-butyrate, and methylene from lipids were obtained in the APS group compared with the VTE group (Figure 3), indicating enhanced glycolysis and ketogenesis by fatty acid catabolism in the APS group. On the other hand, increased concentrations of glycerol, lipids, valine, isoleucine, phenylalanine, and choline in the VTE group compared with the APS group pointed to the importance of altered lipid metabolism and BCAA metabolism in VTE patients. The reported data showed that BCAAs facilitate platelet activation and they are at a high risk for thrombosis [17]. BCAAs are essential amino acids (valine—Val; leucine—Leu; isoleucine—Ile) and they have a key role in the regulation of platelet activation. BCAA catabolism in the presence of multiple enzymes produces branched-chain keto amino acids (BCKA) and acyl-CoA as metabolites [17,20,21]. BCKAs of Val and Ile have a more important role in elevating platelet activation than the keto metabolites of Leu. Moreover, propionyl-CoA is the only metabolite of the BCKAs of Val and Ile and increases platelet activation. Propionyl-CoA, as an intermediate product of Val and Ile catabolism, is a donor molecule for protein propionylation [20,22,23], and an enhancement of the propionylation of cytoskeletal proteins was linked to the upregulation of platelet activation. Also, increased BCAA and fatty acid levels can disturb glycolysis and the TCA cycle by inhibiting the activity of pyruvate dehydrogenase and suppressing the gene expression of succinate dehydrogenase [16,24,25,26]. Finally, these results showed that Val and Ile are associated with the metabolic profile of PAPS and VTE patients with previous thromboembolic episodes. Based on the different levels of the concentrations of glucose, lipids, and BCAAs, these metabolites might be potential biomarkers to differentiate PAPS and VTE patients, including the parameter of aPLs for APS patients.

**Figure 6:**
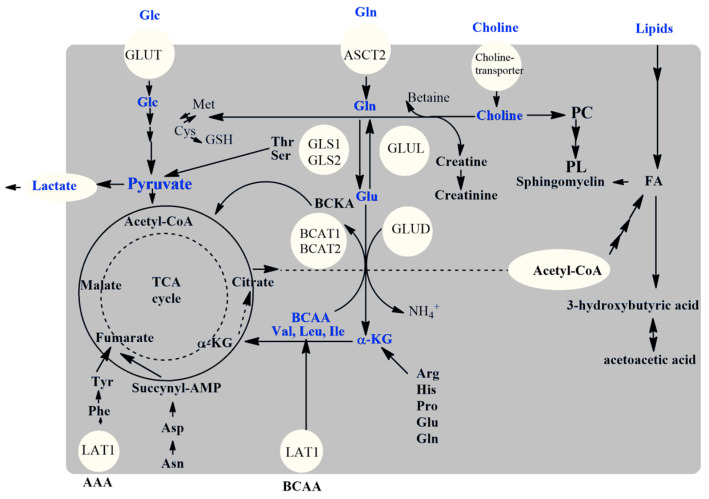
Illustration of the main metabolic pathways reported as altered in VTE and APS: glucose (Glc), glutamine (Gln), glutamate (Glu), α-ketoglutarate (α-KG), choline and phosphatidylcholine (PC), phospholipids (PL), fatty acids (FA), branched-chain amino acids (BCAA, such as valine (Val), leucine (Leu), isoleucine (Ile)), cysteine (Cys), methionine (Met), glutathione (GSH), threonine (Thr), serine (Ser), asparagine (Asn), aspartate (Asp), aromatic amino acids (AAAs, such as phenylalanine (Phe), tyrosine (Tyr)), arginine (Arg), histidine (His), proline (Pro), creatine, creatinine, 3-hydroxybutyric acid, many metabolic enzymes such as glutaminase (GLS1 and GLS2), glutamine synthetase (GLUL), glutamate dehydrogenase (GLUD), branched-chain aminotransferase (BCAT1 and BCAT2), and transporters such as transporter of glucose (GLUT), glutamine (ASCT2), and L-type amino acid transporter 1 (LAT1).

The comparable performance of random forest and XGBoost may suggest that tree-based ensemble methods are particularly suited for this classification task, likely due to their ability to capture nonlinear relationships and interactions between metabolites. The superior ROC AUC of the random forest model suggests it has a better trade-off between sensitivity and specificity, and is more capable of distinguishing among VTE, APS, and HCs. The model’s proficiency in distinguishing HCs from VTE could be attributed to distinct biomarker profiles inherent to the thrombotic state versus normal homeostasis. The lower performance in differentiating VTE from APS indicates that the two conditions share pathophysiological or biomarker similarities, which confounded the model. This overlap stems from the common clinical or biological markers between thrombotic diseases that the model cannot resolve clearly. The equal misclassification rate for APS echoes the complexity of this autoimmune disorder, which manifests a metabolic signature that is not sufficiently distinct from the other conditions, given the available metabolites in this study. In the pathogenesis of thromboembolism and APS, several key players emerge at various stages of the disease. Coagulation factors, inflammatory mediators, and endothelial cells are central to thrombus formation. In the context of APS specifically, antibodies against phospholipid-binding proteins, such as beta-2 glycoprotein I, play a crucial role in promoting an inappropriate clotting cascade. The metabolic underpinnings of venous thromboembolism and antiphospholipid antibody syndrome, as characterized by ^1^H-NMR spectroscopy, have identified a constellation of metabolites that are significantly altered in affected individuals (Figure 6).

This panel, inclusive of glucose, isoleucine, tyrosine, histidine, glutamate, threonine, and 3-hydroxybutyrate, underscores a disruption in both the primary and secondary metabolic pathways that could potentially serve as a metabolic fingerprint for these conditions. The alterations in glucose metabolism suggest a shift towards a prothrombotic state, possibly mediated by the hyperglycemic induction of endothelial dysfunction [27]. Amino acids such as isoleucine, tyrosine, and histidine, which serve as substrates for coagulation factors and immune modulators [28], were found to be perturbed, indicating a probable disruption in the synthesis or degradation of proteins that are central to the coagulation cascade and immune responses. Additionally, the presence of elevated 3-hydroxybutyrate levels points towards a systemic shift in energy metabolism favoring ketogenesis, a state often associated with inflammatory responses. The relevance of these findings is underscored by their alignment with previously reported pathways associated with VTE and APS [29]. Notably, the dysregulation in branched-chain and aromatic amino acid metabolism has been implicated in previous studies, reflecting the multifactorial nature of these diseases that span across coagulation, immune regulation, and inflammation [30]. Our SHAP analysis, aimed at discerning feature importance using machine learning models, revealed that the identified metabolites hold significant predictive value. This emphasizes their potential role in pathophysiological processes.

The limitations of this study include the fact that it is a single-center study, which restricts other populations from being studied. In addition, the variability in the patients’ profiles that occurs over time may not have been considered, since the one-time profile of the individuals was evaluated.

## 4. Materials and Methods

### 4.1. Study Population

The studied population consisted of patients attending the outpatient Clinic of Hemocentro of Unicamp (Campinas, SP, Brazil) between 2009 and 2020. The inclusion criteria of this case–control study were age above 16 years and a previously confirmed arterial thrombosis or VTE episode at any site, including pulmonary embolism (PE). The exclusion criteria were secondary APS; infection; rheumatologic, renal, hepatic, or inflammatory disease; and anti-inflammatory steroid drug use. In addition, the enrolled patients answered a standardized questionnaire during an interview with an authorized researcher/professional. The VTE group was considered unprovoked without a triggering risk factor and defined by the absence of aPL. The APS group included cases of persistently positive aPL, referred to as positive LAC, and of positive IgG or IgM aCL at moderate to high titers (>40 GPL or MPL) or positive (>the 99th percentile) IgG/IgM aβ2GP1 on two occasions, at least 12 weeks apart [3]. All investigated individuals were age- and gender-matched and showed complete clinical information. On the day of enrollment, patients had blood samples taken. The diagnosis of lower limb VTE was established by Doppler ultrasound, and the diagnosis of PE or venous thrombosis in other sites was made by magnetic resonance or angiotomography. Myocardial infarction was diagnosed by ECG and the presence of serologic markers. The diagnosis of ischemic arterial stroke was performed by cerebral magnetic resonance or angiotomography. The controls were healthy individuals selected from the same geographic area and with the same exclusion criteria as the patients, except for previous VTE or arterial thrombosis. aPL was also determined in the controls to rule out asymptomatic APS.

### 4.2. Blood Serum Samples

Samples were obtained from 5 mL of venous blood collected in a dry tube after peripheral venipuncture. Blood was left to clot for 30 min and centrifuged at 4500× g for 15 min at 4 °C, and then the supernatant was carefully separated and aliquoted (250 μL) into cryovials and stored at −80 °C.

### 4.3. Laboratory Evaluation

The detection of aPL was performed for APS diagnosis following the international guidelines from the International Society of Thrombosis and Haemostasis (ISTH) and the Clinical and Laboratory Standard Institute (CLSI) [31,32].

### 4.4. Metabonomics by ^1^H-NMR

The 96 serum samples were thawed at 4 °C, diluted with D2O (99.9%, Cambridge Isotope Laboratories, Inc., Andover, MA, USA, 1/1, *v*/*v*), and centrifuged, and the supernatants were transferred to 5 mm NMR tubes. High-resolution ^1^H-NMR with water signal elimination (noesygppr1d) and ^1^H-NMR spectra with a T_2_ filter (cpmgpr1d) were acquired on the Bruker AVANCE III 600 MHz spectrometer (Billerica, MA, USA) using the inverse triple-core probe (TBI) at 25 °C. Two-dimensional NMR total correlation spectroscopy (TOCSY) spectral data were recorded using the mlevgpphw5 pulse sequence. One- and two-dimensional NMR data, chemical shifts, coupling constants, and the multiplicity of peaks were used for metabolite assignments (see Figure S1 and Table S3 in the Supplementary Information).

### 4.5. Statistical and Machine Learning Analyses

#### 4.5.1. Statistical Analysis Approach

The statistical analyses of the clinical and laboratory data were conducted using GraphPad Prism 5.0 (San Diego, CA, USA) and the Stats package in Python software (3.13). The Shapiro–Wilk test was initially used to assess the distribution of variables. Descriptive statistics, presented as the median and interquartile range (IQR; 25–75%), were calculated for each dataset. The Kruskal–Wallis test facilitated the comparison of three or more unpaired or unmatched groups, with a *p*-value < 0.05 denoting statistical significance. The Mann–Whitney test was employed for analyzing two unmatched groups. The Benjamini–Hochberg false discovery rate (BH-FDR) method corrected the *p*-values, providing q-values where all values below 0.05 were considered significant.

For the ^1^H-NMR spectral analysis, the preprocessing steps included baseline and phase correction, alignment, and referencing. The spectra were categorized into groups, namely healthy controls (HCs, n = 32), antiphospholipid syndrome patients (APS, n = 32), and venous thromboembolism patients (VTE, n = 32), all matched by gender and age. A focused comparison involved matching 8 triple-positivity APS patients with 8 VTE patients for an in-depth analysis. The chemometric analysis, conducted using MetaboAnalyst 5.0, included multivariate analysis (PLS-DA and oPLS-DA), univariate analyses (*t*-test), K-means clustering, and hierarchical cluster analyses after normalization of the spectra. The diagnostic accuracy of the selected metabolites was evaluated using AUCs from receiver operating characteristic curve analyses.

#### 4.5.2. Machine Learning Methodology

A comprehensive suite of machine learning algorithms was employed to classify patients into indicative health status groups (VTE, APS, HCs). The selected algorithms, including ensemble methods (random forest, XGBoost), support vector machines (SVM), and neural networks (multilayer perceptron classifier), were chosen for their ability to capture diverse patterns within the dataset. Random forest (RF) utilized an ensemble of decision trees to improve the model’s robustness and accuracy, reducing overfitting risks and enhancing the generalization of new data. The support vector machine (SVM) found the optimal hyperplane for class separation in high-dimensional spaces, employing a one-vs.-one approach for multiclass classification with probability estimates. The multilayer perceptron classifier (MLP), a neural network, utilized backpropagation for learning, employing the ‘relu’ activation function and the ‘lbfgs’ solver for weight optimization. XGBoost built sequential trees, each correcting the predecessor’s errors, focusing on the model’s weaknesses to create a strong learner.

#### 4.5.3. Performance Evaluation and Model Interpretation

Performance metrics, including the accuracy, AUC score, precision, recall, and F1-score, were employed to evaluate each model’s predictive power comprehensively. A confusion matrix described the best model’s performance on the test data, indicating true positives, false positives, true negatives, and false negatives. SHAP (SHapley Additive exPlanations) analysis was utilized to interpret the machine learning models’ decision-making processes, assessing individual features’ impact on the predictive outcomes. This analysis provided insights into the most influential metabolites in classifying patients into the HC, VTE, and APS groups, enhancing the understanding of the models’ behavior and feature importance.

## 5. Conclusions

This comprehensive NMR-based metabolomic study revealed differences in the metabolic profiles between VTE and HCs, between APS and HCs, and between VTE and triple-positive APS groups. Alterations in the concentrations of metabolites pointed to changed metabolic pathways of glycolysis, the TCA cycle, lipid metabolism, and branched-chain amino acid (BCAA) metabolism in VTE and APS patients. These metabolites might be potential biomarkers to differentiate APS and VTE patients, including the parameter of aPLs for APS patients. The presented approach offers a prospective starting point for exploring and summarizing the complexity of the interrelated metabolites in VTE and APS.

## Figures and Tables

**Table 1:** Meaningful metabolites from amino acid profiling were selected based on the multiple hypothesis comparison with FDR correction.

Metabolite	Direction	Row q-Value	*p*-Value	AUC HCs vs. VTE	AUC HCs vs. APS	AUC VTE vs. APS
Glucose	Decreased	0.00	0.01	0.78	0.69	0.44
Isoleucine	Decreased	0.00	0.04	0.51	0.71	0.72
Tyrosine	Increased	0.00	0.02	0.34	0.23	0.38
Histidine	Increased	0.00	0.01	0.25	0.22	0.54
Glutamate	Increased	0.00	0.00	0.23	0.22	0.52
Threonine	Increased	0.00	0.00	0.19	0.22	0.56
3-Hydroxy butyrate	Increased	0.00	0.00	0.18	0.19	0.58

**Table 2:** Comparative performance metrics of classification algorithms.

	Accuracy	F1-Score	Recall	Precision	ROC AUC
Random forest classifier	0.62	0.62	0.62	0.63	0.82
XGBoost classifier	0.62	0.62	0.62	0.61	0.76
MLP classifier	0.45	0.43	0.45	0.43	0.71
Support vector classifier	0.41	0.40	0.42	0.41	0.72

This table provides a summary of the performance metrics for different machine learning classifiers used in the study. Metrics such as accuracy, F1-score, recall, precision, and the receiver operating characteristic area under the curve (ROC AUC) are presented for the random forest, XGBoost, multilayer perceptron (MLP), and support vector machine (SVM) classifiers. These metrics were calculated based on the multiclass classification of patients into healthy controls (HCs), venous thromboembolism (VTE) patients, and antiphospholipid syndrome (APS) patients.

**Table 3:** Detailed performance metrics of the random forest classifier for each class.

	Precision	Recall	F1-Score
HCs	0.56	0.56	0.56
VTE	0.89	0.80	0.84
APS	0.45	0.50	0.48

This table breaks down the performance of the random forest classifier for each class—HCs, VTE, and APS—in terms of precision, recall, and F1-score. These metrics are crucial for understanding how well the classifier performs for each specific condition.

## Data Availability

The metabonomics data presented in this study are unavailable due to privacy or ethical restrictions.

## Supplementary Materials

The following supporting information can be downloaded at https://www.mdpi.com/article/10.3390/molecules29245895/s1. Figure S1. Examples of ^1^H-NMR data (0.60–5.40 and 6.80–8.60 ppm) from the APS serum group of patients. The spectra were acquired on a Bruker AVANCE III 600 MHz at 25 °C. (a) The spectra were obtained by a ^1^H-NMR pulse sequence with a T_2_ filter (cpmgpr1d). (b) Correlation map for the ^1^H-^1^H NMR TOCSY experiment. The numbers indicate the most essential metabolites identified in this study: 1, -CH_3_ group from lipids (LDL and VLDL); 2, isoleucine; 3, valine; 4, 3-hydroxy-butyrate; 5, -CH_2_ from lipids; 6, glutamate; 7, glutamine; 8, lysine; 9, creatinine; 10, choline; 11, glucose; 12, glycerol; 13, threonine; 14, tyrosine; 15, phenylalanine; and 16, histidine. Figure S2. Univariate receiver operating characteristic (ROC) curve analysis was performed on metabolites distinguishing APS patients from VTE patients. Table S1. Demographic and clinical characteristics of patients with VTE, VTE with APS, and healthy individuals. Table S2. Percentage of antiphospholipid syndrome antibodies. Table S3. Chemical shifts, peak multiplicities, and coupling constants of some of the most essential biomarkers in venous thromboembolism (VTE) and antiphospholipid syndrome (APS) (1–16) as depicted in Figure S1. Table S4. Important metabolites selected by univariate analysis (*t*-tests) were identified as the metabolites with the lowest *p*-value in the ^1^H-NMR CPMG serum data in VTE patients (VTE, n = 32) and healthy controls (HCs, n = 32). Table S5. Important metabolites selected by univariate analysis (*t*-tests) were identified as the metabolites with the lowest *p*-value in the ^1^H-NMR CPMG serum data in antiphospholipid syndrome patients (APS, n = 32) and healthy controls (HCs, n = 32). Table S6. Important metabolites selected by univariate analysis (*t*-tests) were identified as the metabolites with the lowest *p*-value in the ^1^H-NMR CPMG serum data in VTE (n = 8) and antiphospholipid syndrome patients (APS, n = 8).

## References

[1] Ruiz-Irastorza G., Crowther M., Branch W., Khamashta M.A. (2010). Antiphospholipid syndrome. Lancet.

[2] Cervera R., Piette J.C., Font J., Khamashta M.A., Shoenfeld Y., Camps M.T., Jacobsen S., Lakos G., Tincani A., Kontopoulou-Griva I. (2002). Antiphospholipid syndrome: Clinical and immunologic manifestations and patterns of disease expression in a cohort of 1,000 patients. Arthritis Rheum..

[3] Miyakis S., Lockshin M.D., Atsumi T., Branch D.W., Brey R.L., Cervera R., Derkesen R.H.W.M., De Groot P.G., Koike T., Meroni P.L. (2006). International consensus statement on an update of the classification criteria for definite antiphospholipid syndrome (APS). J. Thromb. Haemost..

[4] Funke A., Danowski A., Castro Oliveira de Andrade D., Rêgo J., Abramino Levy R. (2017). The importance of recognizing antiphospholipid syndrome in vascular medicine. J. Vasc. Bras..

[5] Mallhi R.S., Kushwaha N., Chatterjee T., Philip J. (2016). Antiphospholipid syndrome: A diagnostic challenge. Med. J. Armed Forces India.

[6] Sung Y., Spagou K., Kafeza M., Kyriakides M., Dharmarajah B., Shalhoub J., Diaz J.A., Wakefield T.W., Holmes E., Davies A.H. (2018). Deep vein thrombosis exhibits characteristic serum and vein wall metabolic phenotypes in the inferior vena cava ligation mouse model. Eur. J. Vasc. Endovasc. Surg..

[7] Emwas A.H., Roy R., McKay R.T., Tenori L., Saccenti E., Nagana Gowda G.A., Raftery D., Alahmari F., Jaremko L., Jaremko M. (2019). NMR spectroscopy for metabolomics research. Metabolites.

[8] Obi A.T., Stringer K.A., Diaz J.A., Finkel M.A., Farris D.M., Yeomans L., Wakefield T., Myers D.D. (2016). 1D-1H-nuclear magnetic resonance metabolomics reveals age-related changes in metabolites associated with experimental venous thrombosis. J. Vasc. Surg. Venous Lymphat. Disord..

[9] Nicholson J.K., Lindon J.C. (2008). Systems biology: Metabonomics. Nature.

[10] Cao J., Jin Q.Q., Wang G.M., Dong H.L., Feng Y.M., Tian J.S., Yun K.M., Wang Y.Y., Sun J.H. (2018). Comparison of the serum metabolic signatures based on 1H NMR between patients and a rat model of deep vein thrombosis. Sci. Rep..

[11] Deguchi H., Banerjee Y., Trauger S., Siuzdak G., Kalisiak E., Fernández J.A., Hoang L., Tran M., Yegneswaran S., Elias D.J. (2015). Acylcarnitines are anticoagulants that inhibit factor Xa and are reduced in venous thrombosis, based on metabolomics data. Blood.

[12] Escobar M.Q., Tasic L., da Costa T.B.B.C., Stanisic D., Montalvão S., Huber S., Annichino-Bizzacchi J.M. (2021). Serum metabolic profiles based on nuclear magnetic resonance spectroscopy among patients with deep vein thrombosis and healthy controls. Metabolites.

[13] Ballul T., Mageau A., Nicaise P.R., Ajzenberg N., Strukov A., Dossier A., Diane Rouzaud D., Thomas Papo T., Sacré K. (2023). Recurrent thrombotic events after disappearance of antiphospholipid autoantibodies: A long-term longitudinal study in patients with antiphospholipid syndrome. Thromb. Res..

[14] Maekawa K., Sugita C., Yamashita A., Moriguchi-Goto S., Furukoji E., Sakae T., Gi T., Hirai T., Asada Y. (2019). Higher lactate and purine metabolite levels in erythrocyte-rich fresh venous thrombus: Potential markers for early deep vein thrombosis. Thromb. Res..

[15] Franczyk B., Gluba-Brzózka A., Ławiński J., Rysz-Górzyńska M., Rysz J. (2021). Metabolomic profile in venous thromboembolism (VTE). Metabolites.

[16] Ye Z., Wang S., Zhang C., Zhao Y. (2020). Coordinated modulation of energy metabolism and inflammation by branched-chain amino acids and fatty acids. Front. Endocrinol..

[17] Xu Y., Jiang H., Li L., Chen F., Liu Y., Zhou M., Wang J., Jiang J., Li X., Fan X. (2020). Branched-chain amino acid catabolism promotes thrombosis risk by enhancing tropomodulin-3 propionylation in platelets. Circulation.

[18] Zhang L., Li Y., Lin X., Jia C., Yu X. (2019). Liquid Chromatography/Mass Spectrometry based serum metabolomics study on recurrent abortion women with antiphospholipid syndrome. PLoS ONE.

[19] Quintero M., Montalvão S.A.D.L., Tasic L., Huber S.C., Annichino-Bizzacchi J.M. (2019). Comparison of the serum metabolic signatures based on ^1^H NMR between thrombotic antiphospholipid syndrome (APS) patients and healthy individuals. Blood.

[20] Lynch C.J., Adams S.H. (2014). Branched-chain amino acids in metabolic signaling and insulin resistance. Nat. Rev. Endocrinol..

[21] White P.J., McGarrah R.W., Grimsrud P.A., Tso S.C., Yang W.H., Haldeman J.M., Grenier-Larouche T., An J., Lapworth A.L., Astapova I. (2018). The BCKDH kinase and phosphatase integrate BCAA and lipid metabolism via the regulation of ATP-citrate lyase. Cell Metab..

[22] Kebede A.F., Nieborak A., Shahidian L.Z., Le Gras S., Richter F., Gómez D.A., Baltissen M.P., Meszaros G., De Fatima Magliarelli H., Taudt A. (2017). Histone propionylation is a mark of active chromatin. Nat. Struct. Mol. Biol..

[23] Garrity J., Gardner J.G., Hawse W., Wolberger C., Escalante-Semerena J.C. (2007). N-lysine propionylation controls the activity of propionyl-CoA synthetase. J. Biol. Chem..

[24] Li T., Zhang Z., Kolwicz S.C., Abell L., Roe N.D., Kim M., Zhou B., Cao Y., Ritterhoff J., Gu H. (2017). Defective branched-chain amino acid catabolism disrupts glucose metabolism and sensitizes the heart to ischemia-reperfusion injury. Cell Metab..

[25] Wang J., Liu Y., Lian K., Shentu X., Fang J., Shao J., Chen M., Wang Y., Zhou M., Sun H. (2019). BCAA catabolic defect alters glucose metabolism in lean mice. Front. Physiol..

[26] Savage D.B., Petersen K., Shulman G.I. (2007). Disordered lipid metabolism and the pathogenesis of insulin resistance. Physiol. Rev..

[27] Esper R., Vilariño J., Machado R., Paragano A. (2008). Endothelial Dysfunction in Normal and Abnormal Glucose Metabolism. Adv. Cardiol..

[28] Adams T.E., Huntington J.A. (2006). Thrombin-cofactor interactions: Structural insights into regulatory mechanisms. Arterioscler. Thromb. Vasc. Biol..

[29] Nayak L., Sweet D.R., Thomas A., Lapping S.D., Kalikasingh K., Madera A., Vinayachandran V., Padmanabhan R., Vasudevan N.T., Myers J.T. (2022). A targetable pathway in neutrophils mitigates both arterial and venous thrombosis. Sci. Transl. Med..

[30] Rahnavard A., Mann B., Giri A., Chatterjee R., Crandall K.A. (2022). Metabolite, protein, and tissue dysfunction associated with COVID-19 disease severity. Sci. Rep..

[31] Clinical and Laboratory Standards Institute (2014). H60-A Laboratory Testing for the Lupus Anticoagulant; Approved Guideline.

[32] Devreese K.M.J., Ortel T.L., Pengo V., de Laat B. (2018). Laboratory criteria for antiphospholipid syndrome: Communication from the SSC of the ISTH. J. Thromb. Haemost..

