# Supplementary material for: Mapping Thrombosis Serum Markers by 1H-NMR Allied with Machine Learning Tools
Source: Molecules. 2024 Dec 13;29(24):5895. doi: 10.3390/molecules29245895 (PMC11676712; doi:10.3390/molecules29245895)
Supplement: Supplementary file 1 [file molecules-29-05895-s001.zip › molecules-3313289-supplementary.pdf]

Supplementary Information

Mapping Thrombosis Serum Markers by ^{1}H-NMR allied with Machine Learning Tools

Lucas G. Martins[1], Bruna M. Manzini[2], Silmara Montalvão[2], Millene A. Honorato[2], Marina P. Colella[2], Gabriela G. Y. Hayakawa[2], Erich V. de Paula[2], Fernanda Orsi[2], Erik S. Braga[1], Nataša Avramović[3], Folorunsho Bright Omage[1], Ljubica Tasic[1,*] and Joyce M Annichino-Bizzacchi[2,*]

[1] Laboratory of Biological Chemistry, Department of Organic Chemistry, Institute of Chemistry, Universidade Estadual de Campinas, Campinas, Sao Paulo, Brazil. Zip code: 13083-970; lgmartins1984@gmail.com; e264601@dac.unicamp.br; bright@unicamp.br; ljubica@unicamp.br

[2] Laboratory of Hemostasis, HEMOCENTRO-UNICAMP, Universidade Estadual de Campinas, Campinas, Sao Paulo, Brazil. Zip code: 13083-878; brunamanzini@gmail.com; silmara@unicamp.br; millene.ealhonorato@gmail.com; marinasp@unicamp.br; hayakawa@unicamp.br; erich@unicamp.br; ferorsi@unicamp.br; joyce@unicamp.br

[3] University of Belgrade - Faculty of Medicine, Institute of Medical Chemistry, Belgrade, Serbia. Zip code: 11000; natasa.avramovic@med.bg.ac.rs

* Correspondence: Ljubica Tasic and Joyce M Annichino-Bizzacchi. E-mail: ljubica@unicamp.br (L. T.); Tel.: +55 19 3521 1106 and 98137 7561 and joyce@unicamp.br (J. M. A. -B.); Tel.: +55 19 3521 8755

Table S1. Demographic and clinical characteristics of patients with VTE, VTE with APS, and Healthy Individuals

Demographic, clinical, and laboratory characteristics of enrolled participants				
**Characteristic**		**VTE (n = 32)**	**APS (n = 32)**	**HC (n = 32)**
Age (y), median (IQR)		44.50 (33.25 – 55.50)	53.50 (34.75 – 64.75)	50.50 (33.25 – 61.25)
Gender	Male, n (%)	10 (32.3)	20 (62.5)	13 (40.6)
	Female, n (%)	22 (68.8)	12 (37.5)	19 (59.4)
Ethnic origin	Caucasian, n (%)	22 (68.8)	24 (75.0)	28 (87.4)
	Afrodescendant, n (%)	9 (28.1)	5 (15.6)	4 (12.5)
	Indigenous, n (%)	1 (3.1)	2 (6.3)	0 (0)
	Asian, n (%)	0 (0)	1 (3.1)	0 (0)
BMI, median (IQR)		30.65 (26.45 - 35.90)	27.40 (23.33 - 31.18)	28.05 (25.55 - 32.10)
Comorbidities	Overweight (25.0 < BMI < 29.9), n (%)	6 (18.8)	6 (18.8)	18 (53.3)
	Obesity (BMI ≥ 30.0), n (%)	15 (46.9)	7 (21.9)	11 (18.8)
	Hypertension, n (%)	12 (32.5)	13 (40.6)	10 (31.3)
	Dyslipidemia, n (%)	9 (28.1)	12 (32.5)	3 (9.4)
	Diabetes, n (%)	10 (31.3)	3 (9.4)	4 (12.5)
Medication	Statin, n (%)	6 (18.8)	12 (32.5)	0 (0)
	Antihypertensive, n (%)	11 (18.8)	13 (40.6)	7 (21.9)
	Diabetes drug, n (%)	7 (21.9)	3 (9.4)	3 (9.4)
	Acetylsalicylic acid, n (%)	8 (25.0)	9 (28.1)	2 (6.3)
	Antidepressant, n (%)	2 (6.3)	2 (6.3)	2 (6.3)
	Anti Inflammatory, n (%)	0 (0)	1 (3.1)	0 (0)
	Analgesic, n (%)	0 (0)	1 (3.1)	0 (0)
	Others, n (%)	11 (34.4)	22 (68.8)	8 (25.0)

Venous thrombosis (VTE n=31, APS n=28)	Age at diagnosis (y), median (IQR)	45.0 (34.00 – 56.00)	54.50 (39.75 – 66.50)	NA
	Provoked, n (%)<br>Major Transient<br>Minor Transient<br>Persistent	20 (64.5)<br>5 (25%)<br>13 (65%)<br>2 (10%)	7 (25)<br>1 (14.3%)<br>5 (71.4%)<br>1 (14.3%)	NA
	Unprovoked, n (%)	11 (35.5)	21 (75)	NA
	History of previous VTE, n (%)	0 (0)	6 (21.4)	NA
	Time between diagnosis and blood sample collection (m), median (IQR)	26.50 (17.00 – 45.50)	25.00 (15.50 – 94.00)	NA
Arterial thrombosis (VTE n=01, APS n=04)	Age at diagnosis (y), median (IQR)	16.00	25.50 (22.00– 42.50)	NA
	History of previous VTE, n (%)	0 (0)	0 (0)	NA
	Time between diagnosis and blood sample collection (m), median (IQR)	27.50 (15.00 – 192.00)	25.00 (16.00 – 224.00)	NA
Anticoagulation	Warfarin, n (%)	20 (62.5)	29 (90.6)	NA
	Rivaroxaban, n (%)	9 (28.1)	3 (9.4)	NA
	Without anticoagulation therapy, n (%)	3 (9.4)	1 (3.1)	NA
VTE occurrence site	Distal right lower limb, n (%)	2 (6.3)	1 (3.1)	NA
	Proximal right lower limb, n (%)	11 (34.4)	5 (15.6)	NA
	Distal left lower limb, n (%)	3 (9.4)	5 (15.6)	NA
	Proximal left lower limb, n (%)	8 (25.0)	9 (28.1)	NA
	PE, n (%)	14 (43.8)	7 (21.9)	NA

Cerebral vein thrombosis, n (%)	0 (0)	2 (6.3)	NA
Retinal vein occlusion, n (%)	1 (3.1)	3 (9.4)	NA

Table S2. Percentage of antiphospholipid syndrome antibodies

Antibody APS	APS (n = 32)
LAC (Lupus Anticoagulant)	28 (87.5%)
LAC and anti-beta 2 glycoprotein I	12 (37.5%)
aCL (anticardiolipin)	7 (21.9%)
Triple positive	8 (25.0%)

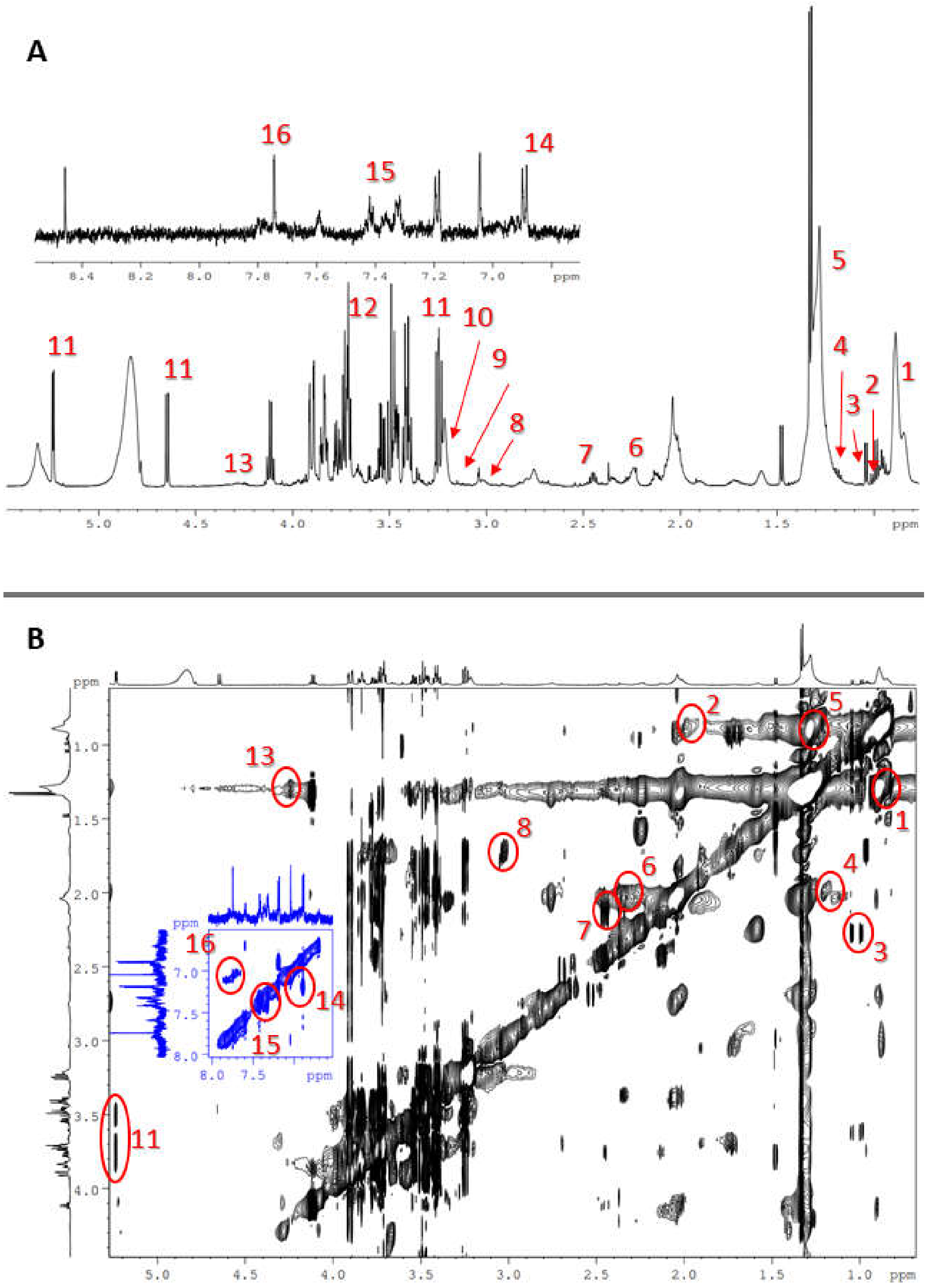

Figure S1. Examples of ^{1}H-NMR data (0.60-5.40 ppm, and 6.80-8.60 ppm) from the APS serum group of patients. The spectra were acquired on the Bruker AVANCE III 600 MHz at 25 °C. (**a**) The spectrum was obtained by a ^{1}H-NMR pulse sequence with a T_2 filter (cpmgpr1d). (**b**) Correlation map for ^{1}H-^{1}H NMR TOCSY experiment. The numbers indicate the most essential metabolites identified in this study: 1, $-CH_3$ group from lipids (LDL and VLDL); 2, isoleucine; 3, valine; 4, 3-hydroxy-butyrate; 5, $-CH_2$ from lipids; 6, glutamate; 7, glutamine; 8, lysine; 9, creatinine; 10, choline; 11, glucose; 12, glycerol; 13, threonine; 14, tyrosine; 15, phenylalanine; and 16, histidine.

Table S3. Chemical shifts, peak multiplicities, and coupling constants of some of the most essential biomarkers in venous thromboembolism (VTE) and antiphospholipid syndrome (APS) (1-16) as depicted in Figure S1.

Metabolite	Nº	Chemical shift (ppm), peaks multiplicities, and coupling constants
**CH_3 from lipids**	1	From 0.78 to 0.92
**Isoleucine**	2	0.98 t (*J* = 8.0 Hz); 1.01 d (*J* = 9.0 Hz); 1.25 m; 1.46 m; 1.97 m; 3.66 d (*J* = 4.0 Hz)
**Valine**	3	0.99 d (*J* = 8.0 Hz); 1.04 d (*J* = 7.0 Hz); 2.26 m; 3.64 d (*J* = 4.0 Hz)
**3-Hydroxybutyrate**	4	1.20 d (*J* = 6.3 Hz); 2.34 m; 2.40 m; 4.13 m
**CH_2 in lipids**	5	From 1.20 to 1.40
**Glutamate**	6	2.04 m; 2.12 m; 2.34 m; 3.76 dd (*J* = 7.2, 4.7 Hz)
**Glutamine**	7	2.07 m; 2.45 m; 3.76 t (*J* = 6.2 Hz)
**Lysine**	8	1.46 m; 1.71 m; 1.89 m; 3.02 t; 3.74 t (*J* = 6.1 Hz)
**Creatinine**	9	3.04 s; 3.92 s
**Choline**	10	3.21 s; 3.55 dd (*J* = 5.8, 4.2 Hz); 4.14 m
**Glucose**	11	3.23 dd (*J* = 9.4, 8.0 Hz); 3.40 m; 3.46 m; 3.52 dd (*J* = 9.8, 3.8 Hz); 3.73 m; 3.82 m; 3.88 dd (*J* = 12.3, 2.2 Hz); 4.63 d (*J* = 8.0 Hz); 5.22 d (*J* = 3.8 Hz); or 3.25 m; 3.49 m; 3.50 m; 3.88 m; 3.91 m; 4.66 d (*J* = 3.8 Hz)
**Glycerol**	12	3.55 m; 3.66 m; 3.80 m
**Threonine**	13	1.32 d (*J* = 6.7 Hz); 3.57 (*J* = 6.5 Hz); 4.24 m
**Tyrosine**	14	3.03 dd (*J* = 14.6, 8.0 Hz); 3.34 dd (*J* = 14.5, 4.7 Hz); 4.04 dd (*J* = 8.0, 4.7 Hz); 6.89 d (*J* = 8.4 Hz); 7.19 d (*J* = 8.0 Hz)
**Phenylalanine**	15	3.19 m; 3.98 dd (*J* = 7.9, 5.3 Hz); 7.32 d (*J* = 7.0 Hz); 7.32 m; 7.37 m; 7.42 m

**Histidine**	16	3.16 dd (J = 15.6, 7.8 Hz); 3.23 dd (J = 16.1, 4.9 Hz); 3.98 m; 7.04 d (J = 1.1 Hz); 7.75 d (J = 1.1 Hz);

Table S4. Important metabolites selected by Univariate Analysis (t-tests, were identified as the metabolites with the lowest *p*-value in the ^{1}H-NMR CPMG serum data in VTE patients (VTE, n = 32) and healthy control (HC, n = 32) individuals.

**Metabolites**	**t-Test**	***p*-value**	**$-\log_{10}(p)$**
Valine	-4.42	3.99×10^{-5}	4.40
Tyrosine	-4.59	2.25×10^{-5}	4.65
Threonine	-4.33	5.55×10^{-5}	4.25
Lysine	3.94	20.53×10^{-5}	3.69
Histidine	-4.30	6.05×10^{-5}	4.22
Glutamine	4.53	2.79×10^{-5}	4.55
Glucose	3.97	19.11×10^{-5}	3.72
Creatinine	5.70	0.04×10^{-5}	6.45

Table S5. Important metabolites selected by Univariate Analysis (t-tests) were identified as the metabolites with the lowest *p*-value in the ^{1}H-NMR CPMG serum data in antiphospholipid syndrome patients (APS, n = 32) and health control (HC, n = 32) individuals .

**Metabolites**	**t-Test**	***p*-value**	**$-\log_{10}(p)$**
Valine	-5.36	1.29×10^{-5}	5.89
Threonine	3.26	182.85×10^{-5}	2.74
Lysine	-4.89	75.29×10^{-5}	5.12
Isoleucine	3.35	138.33×10^{-5}	2.86
Glutamine	-3.79	34.16×10^{-5}	3.47
CH_2 lipids	-3.48	93.20×10^{-5}	3.03

Table S6. Important metabolites selected by Univariate Analysis (t-tests, were identified as the metabolites with the lowest *p*-value in the ^{1}H-NMR CPMG serum data in VTE (n = 8) and antiphospholipid syndrome patients (APS, n = 8).

**Metabolites**	**t-test**	***p*-value**	**$-\log_{10}(p)$**
Valine	-2.27	0.0395	1.40
Tyrosine	3.12	0.0076	2.12
Phenylalanine	-3.56	0.0032	2.50
Lysine	2.23	0.0425	1.37
Lipids	-2.66	0.0187	1.73
Isoleucine	-2.18	0.0468	1.33
Glycerol	-2.48	0.0266	1.58
Glutamate	2.53	0.0239	1.62
Glucose	2.87	0.0122	1.91
Choline	-2.25	0.0409	1.39
3-Hydroxy-butyrate	2.82	0.0137	1.86
$(CH_2)CH_2CO$ lipids	2.79	0.0144	1.84

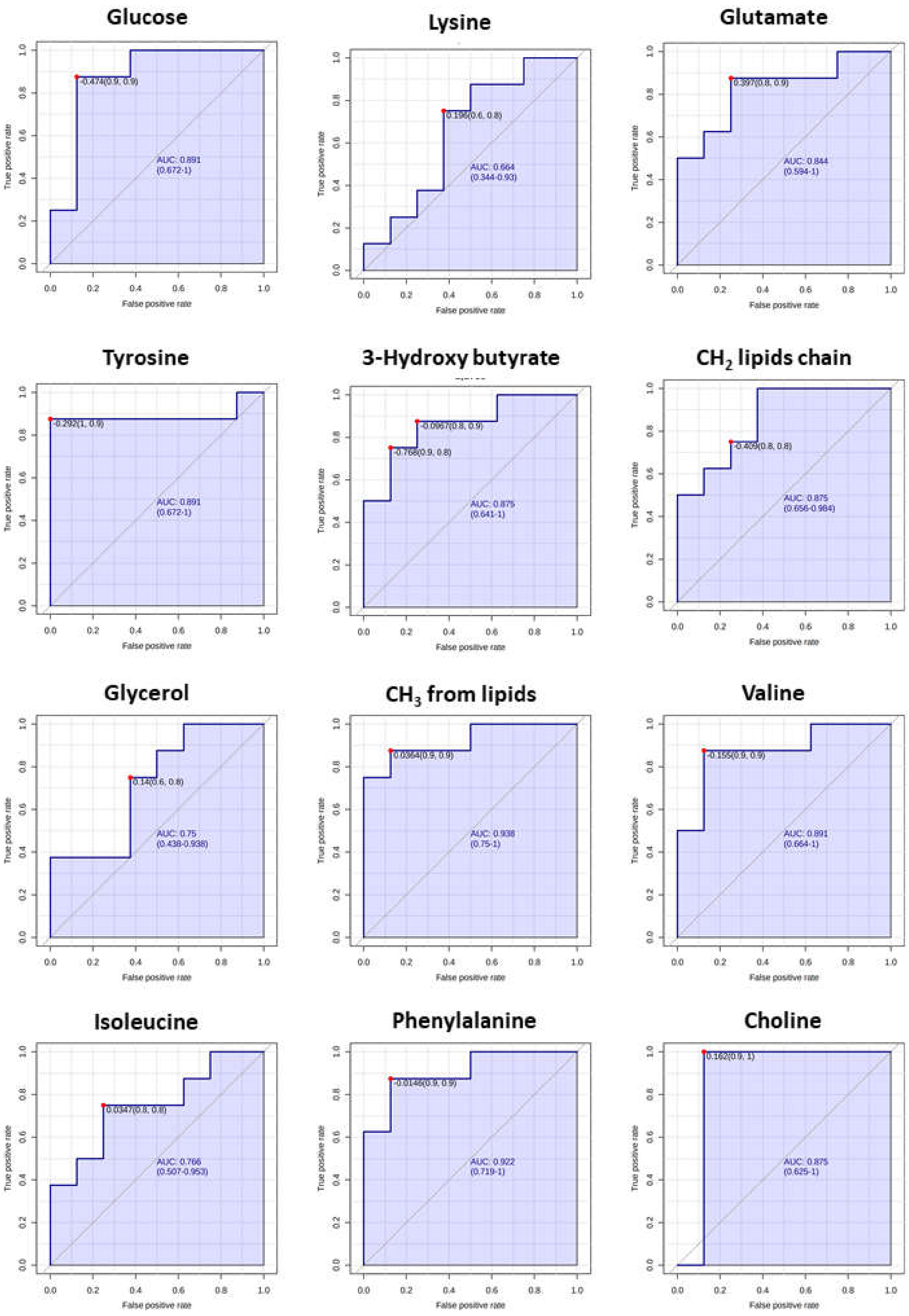

Figure S2. Univariate Receiver Operating Characteristic (ROC) curve analysis was performed on metabolites distinguishing APS patients from VTE patients.
